# Analysis of transient phosphorylation-dependent protein-protein interactions in living mammalian cells using split-TEV

**DOI:** 10.1186/1472-6750-8-55

**Published:** 2008-07-13

**Authors:** Michael C Wehr, Lisa Reinecke, Anna Botvinnik, Moritz J Rossner

**Affiliations:** 1 Research Group 'Gene Expression', Max-Planck-Institute of Experimental Medicine, 37075 Göttingen, Germany; 2 Apoptosis and Proliferation Control Laboratory, Cancer Research UK, London Research Institute (LRI), UK

## Abstract

**Background:**

Regulated protein-protein interactions (PPIs) are pivotal molecular switches that are important for the regulation of signaling processes within eukaryotic cells. Cellular signaling is altered in various disease conditions and offers interesting options for pharmacological interventions. Constitutive PPIs are usually mediated by large interaction domains. In contrast, stimulus-regulated PPIs often depend on small post-translational modifications and are thus better suited targets for drug development. However, the detection of modification-dependent PPIs with biochemical methods still remains a labour- and material-intensive task, and many pivotal PPIs that are potentially suited for pharmacological intervention most likely remain to be identified. The availability of methods to easily identify and quantify stimulus-dependent, potentially also transient interaction events, is therefore essential. The assays should be applicable to intact mammalian cells, optimally also to primary cells in culture.

**Results:**

In this study, we adapted the split-TEV system to quantify phosphorylation-dependent and transient PPIs that occur at the membrane and in the cytosol of living mammalian cells. Split-TEV is based on a PPI-induced functional complementation of two inactive TEV protease fragments fused to interaction partners of choice. Genetically encoded transcription-coupled and proteolysis-only TEV reporter systems were used to convert the TEV activity into an easily quantifiable readout. We measured the phosphorylation-dependent interaction between the pro-apoptotic protein Bad and the adapter proteins 14-3-3ε and ζ in NIH-3T3 fibroblasts and in primary cultured neurons. Using split-TEV assays, we show that Bad specifically interacts with 14-3-3 isoforms when phosphorylated by protein kinase Akt-1/PKB at Ser136. We also measured the phosphorylation-dependent Bad/14-3-3 interactions mediated by endogenous and transient Akt-1 activity. We furthermore applied split-TEV assays to measure the phosphorylation-dependent interactions of Neuregulin-1-stimulated ErbB4 receptors with several adapter proteins.

**Conclusion:**

Split-TEV assays are well suited to measure phosphorylation-dependent and transient PPIs that occur specifically at the membrane and in the cytosol of heterologous and primary cultured mammalian cells. Given the high sensitivity of the split-TEV system, all assays were performed in multi-plate formats and could be adapted for higher throughput to screen for pharmacologically active substances.

## Background

Constitutive and regulated PPIs are the main organizing principles within signaling cascades the integration of which results in an adaptive cellular behaviour. Modification-dependent PPIs are often positioned at pivotal positions within signaling pathways, and are thus central to signaling processes at the membrane and in the cytosol of living mammalian cells [1]. Phosphorylation of specific serine or threonine residues by kinases represents the prototype and most abundant type of post-translational protein modifications [2]. In light of the fact that cellular signaling is altered in many disease conditions, functional subunits of signaling processes are the focus of intense research, since they represent attractive targets for pharmacological intervention [3]. In contrast to constitutive PPIs, stimulus-regulated PPIs often depend on small post-translational modifications, and are thus better suited targets for drug development [4]. However, the detection of modification-dependent PPIs with biochemical methods still remains a labour- and material-intensive task, and many pivotal PPIs potentially suited for pharmacological perturbation most likely still remain to be identified. Therefore, the availability of methods to easily screen and identify stimulus-dependent, potentially transient, interaction events is essential. Ideally, the assays should be applicable to intact mammalian cells, including cultured primary cells.

Recently, we reported the development of the split-TEV approach that allowed us to monitor the ligand-induced dimerisation of ErbB receptors at the membrane of mammalian cells [5]. Split-TEV is based on the functional complementation of two inactive TEV protease fragments fused to interacting proteins. The PPI-dependent TEV protease activity can be followed by several reporters, which either rely on a fluorescent or a luminescent readout [5]. In this study, we wanted to adapt the split-TEV system to analyse constitutive and phosphorylation-dependent interactions of full-length proteins that occur in the cytosol and at the membrane. For the technical proof-of-principle for cytosolic interactions, we chose the interactions between Bad and 14-3-3 isoforms as a model system [6,7]. Both, the 14-3-3 and Bad proteins are involved in the regulation of apoptosis and survival signaling [8,9]. Bad is a pro-apoptotic protein exerting its action by binding to the anti-apoptotic, mitochondrially localised proteins Bcl-X_L _and Bcl2, thereby inactivating the Bcl proteins [10]. However, upon phosphorylation at serine 136 by protein kinase Akt-1/PKB, Bad can be complexed by 14-3-3 proteins in the cytosol, thus preventing the association with the Bcl proteins and inhibiting apoptosis [6,11]. 14-3-3 proteins were shown to be involved in sequestering functions through binding to phoshorylated proteins and consequently influencing signaling events [9,11]. There are seven 14-3-3 genes giving rise to the seven isoforms β, γ, ε, η, σ, τ (or θ) and ζ. The 14-3-3 isoforms can functionally compensate for each other, but can also mediate specific cellular functions: the σ isoform for example is implicated in cancer and cell cycle regulation [9,12], whereas the isoforms ε and ζ are highly expressed in postmitotic cells of the brain [13]. Additionally, 14-3-3 proteins can homo- and heterodimerise [9].

To demonstrate the applicability of the split-TEV system to analyse phosphorylation-dependent interactions at the membrane, we chose stimulus-dependent interactions of the ErbB4 receptor with various cytosolic adapter proteins. ErbB4 belongs to the family of ErbB receptor tyrosine kinases, which are involved in diverse signaling mechanisms ranging from proliferation to differentiation and neuronal specification [14-16]. Upon ligand binding, ErbB4 homo- or heterodimerises, followed by an autophosphorylation *in trans*, which then leads to the recruitment of SH2 domain-containing adaptor proteins, such as Grb2, Shc1 and the regulatory subunit of PI3K (PI3Kp85) [20,21]. Neuregulin-1 (Nrg1) represents the best characterized ErbB4 ligand and has been shown to be implicated several diseases, including cancer and schizophrenia [17-19].

In this report, we measured the homo- and heterodimeric interactions of 14-3-3 isoforms and the modification-dependent interaction between full-length cytosolic Bad and 14-3-3 isoforms ε and ζ in heterologous NIH-3T3 cells and primary neurons using the split-TEV system. Moreover, we measured the Nrg1-induced interactions of several SH2-adapter proteins with phosphorylated ErbB4 in living cells.

## Results

### Split-TEV reporters to monitor interactions of cytoplasmic proteins

**Figure 1:**
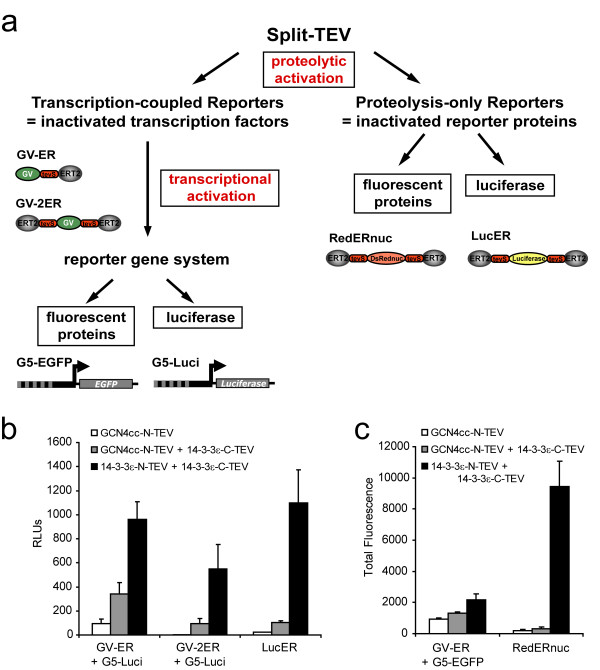
**Analysis of cytosolic split-TEV reporters to monitor the dimerization of 14-3-3 proteins**. (a) The flow-chart depicts the transcription-coupled reporter systems (GV-ER/GV-2ER used in combination with a GV-dependent reporter construct) and the proteolysis-only reporter systems (RedERnuc/LucER). Whereas the readout via the transcription-coupled reporter systems is generated by two steps, namely proteolytic cleavage and transcriptional activation, the proteolysis-only reporter systems require the proteolytic activation only. Transcription-coupled reporters are inactivated transcription factors; proteolysis-only reporters are inactivated final reporter proteins. tevS, TEV protease cleavage site; GV, artificial transcription factor composed of the DNA-binding domain of Gal4 from yeast and the transactivation domain VP16 from herpes simplex virus; DsRednuc, nuclear variant of DsRed; Luciferase, firefly luciferase moiety; ERT2, modified ligand binding domain of the estrogen receptor. (b, c) Luciferase (a) and FACS (b) assays showing the dimerization of the 14-3-3ε isoform with all cytosolic reporters. NIH-3T3 cells were transfected with the indicated combinations of constructs. GCN4cc-N-TEV was used as a control.

Protein-protein interactions can be measured using a protein complementation approach that is based on TEV protease and is termed split-TEV [5]. In this system, inactive TEV protease fragments are fused to potentially interacting proteins. Upon interaction the reconstituted functional protease activates genetically encoded TEV-specific reporters [5]. The cytosolic reporters used are either proteolysis-only reporters requiring only one step of activation (LucER, RedERnuc) or transcription-coupled reporters with a two-step activation, which consist of the proteolytic activation of the previously silent transcriptional activator GV and the transcriptional activation of a final reporter gene (firefly luciferase or EGFP) (Fig. 1a). The functionality of all cytosolic reporters relies on the modified ligand binding domain of the estrogen receptor, termed ERT2 [22]. For LucER, this domain substantially decreases the biological activity of the luciferase unit, and for GV-2ER, GV is trapped in the cytosol, unless both ERT2 domains are cleaved off [5]. Using both types of reporters, we analyzed the modification dependent interaction of Bad and 14-3-3, as well as the constitutive association of 14-3-3 isoforms.

**Figure 2:**
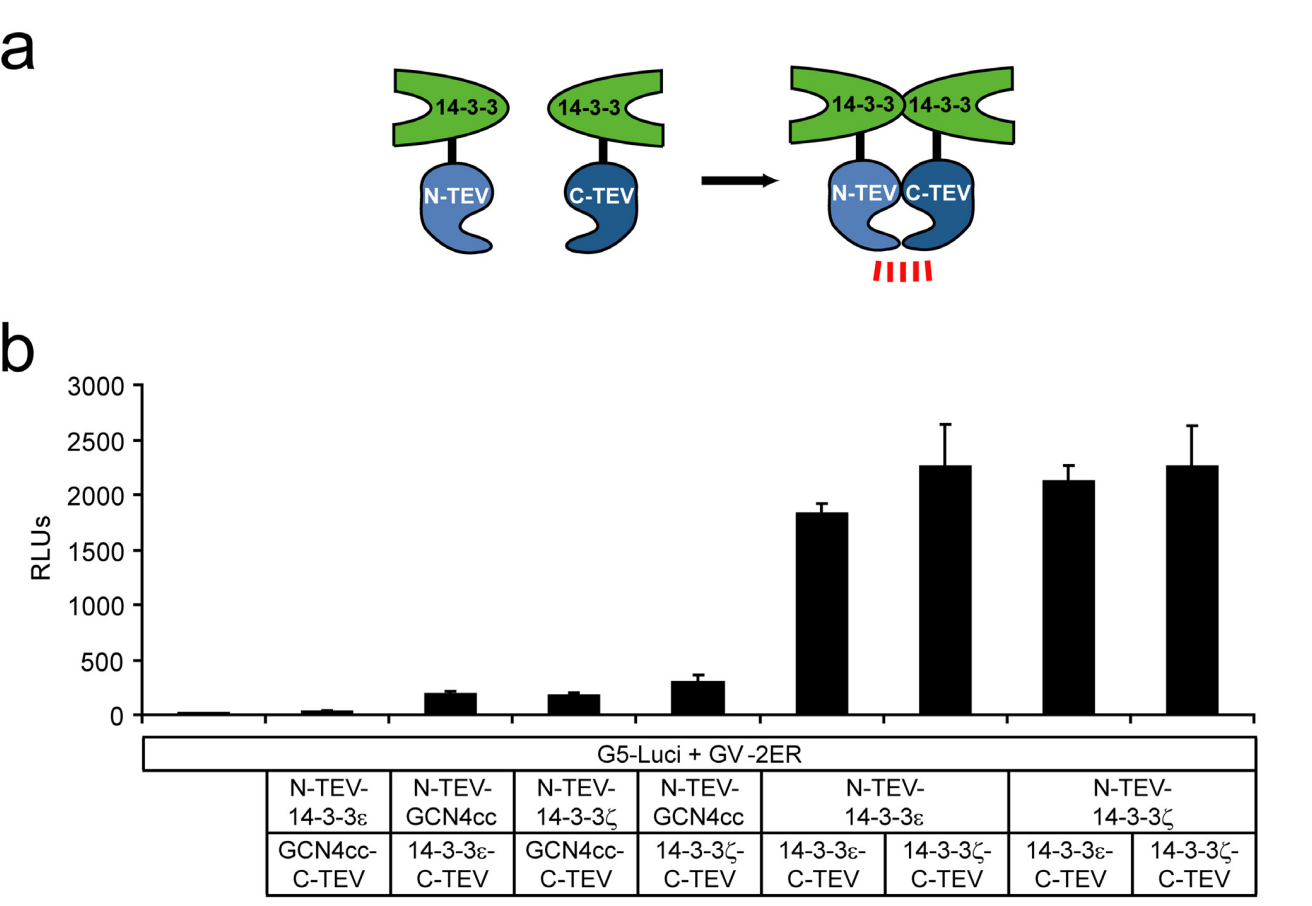
**Homo- and heterodimerization of 14-3-3 isoforms**. (a) Schematic representation of a 14-3-3 split-TEV assay. 14-3-3 isoforms were fused to N-TEV and C-TEV fragments. Upon homo- or heterodimeric interaction the TEV protease activity is reconstituted. (b) Murine 14-3-3 isoforms ε and ζ form homo- and heterodimers. Luciferase readings also verify the flexible usage of TEV fragments. GCN4cc domains were used as negative controls. NIH-3T3 cells were transiently transfected with the constructs as indicated. RLUs, relative luciferase units, n = 6.

For 14-3-3 proteins, homo- and heterodimer formation has been demonstrated [23]. We first performed split-TEV assays to monitor 14-3-3ε homodimerisation using the complete set of transcription-coupled and proteolysis-only reporters with luciferase-based (Fig. 1b) and fluorescent readouts (Fig. 1c). Each reporter indicated a specific interaction of 14-3-3 dimers when compared to control assays. These control assays contained constructs coding for the coiled-coil domain from the GCN4 protein (Fig. 1b, c). With respect to the signal-to-noise ratio, the doubly ERT2-flanked reporter RedERnuc performed best, followed by GV-2ER and LucER. We used GV-2ER and LucER for most experiments, since luciferase assays are less labour-intensive compared to quantitative fluorescence analysis. Next, we examined the homo- and heterodimerisation of the 14-3-3 isoforms ε and ζ. Here, the N-TEV fragments were either N- or C-terminally fused to the 14-3-3 protein isoforms (Fig. 2a). All combinations displayed a comparable degree of activation and we did not observe reduced signals that may have been caused by structural constraints of the differently tagged fusion proteins. Using split-TEV assays with the GV-2ER reporter, we were able to measure the constitutive association between 14-3-3ε and ζ in all possible combinations (Fig. 2b). Corresponding assays with LucER showed similar results (data not shown).

### Monitoring Akt-1 dependent interactions of Bad and 14-3-3 in NIH-3T3 fibroblasts and pimary cultured cortical neurons

**Figure 3:**
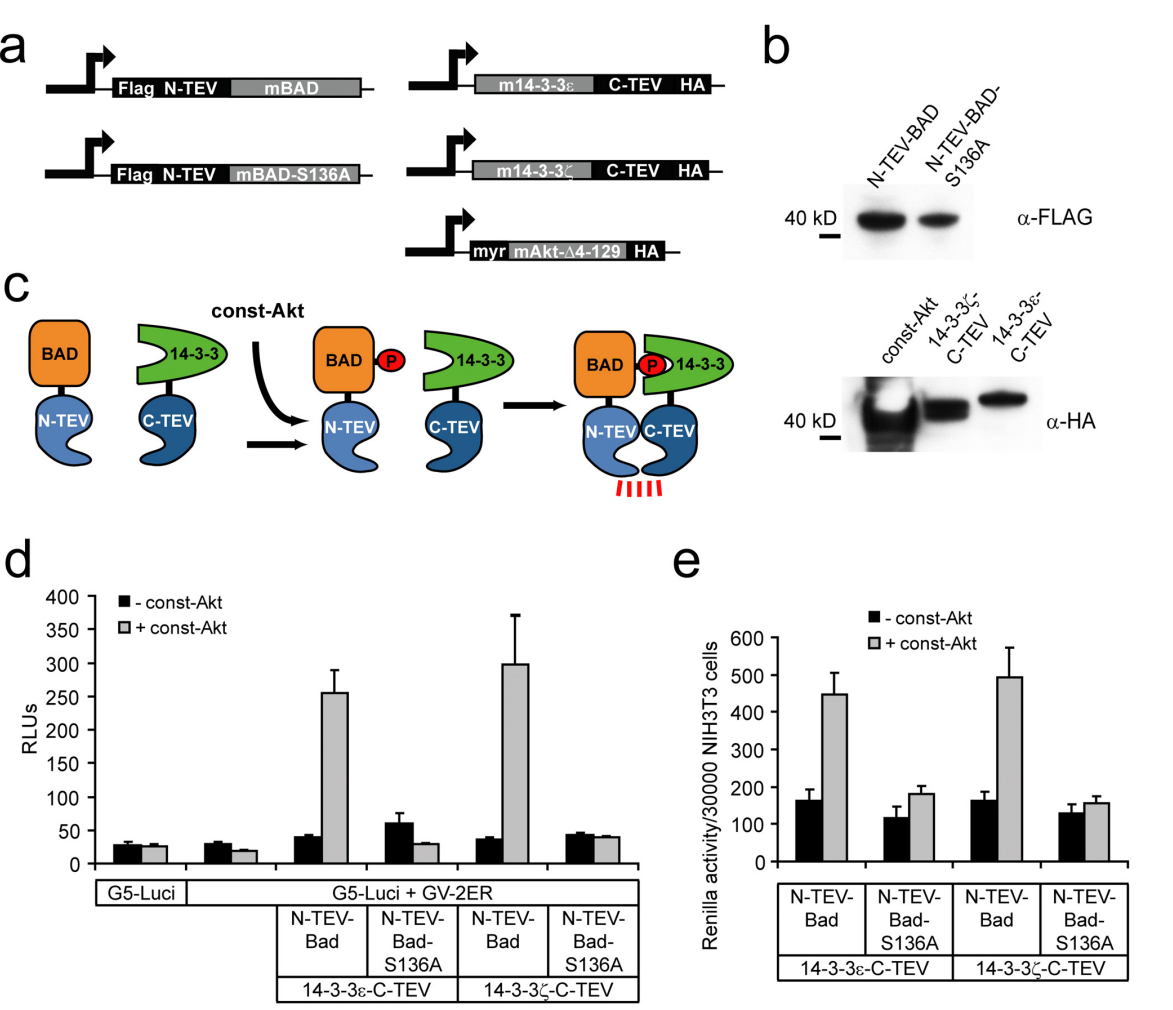
**Quantifying phosphorylation dependent interactions between Bad and 14-3-3 isoforms**. (a) CMV-driven expression constructs are depicted as bar graphs. Murine Bad, mutated Bad-S136A and murine 14-3-3 isoforms ε and ζ were fused to N-terminal or C-terminal TEV fragments as indicated. N-terminal and C-terminal TEV fusions contain a Flag-tag or an HA-tag, respectively. The constitutive form of murine Akt-1 (myr-Akt-Δ4-129-HA) lacks the PH domain, but contains an N-terminal myristoylation signal and a C-terminal HA-tag. (b) Western blot analysis of expression constructs shown in (a). Calculated protein sizes are: N-TEV-Bad, 41.0 kDa; N-TEV-Bad-S136A, 41.0 kDa; Const-Akt, 43.4 kDa; 14-3-3ζ-C-TEV, 42.8 kDa; 14-3-3ε-C-TEV, 44.2 kDa. (c) Principle of TEV fragment fusion proteins used to monitor the phosphorylation dependent interaction of Bad and 14-3-3. When Akt-1 kinase activity is present, Bad becomes phosphorylated enabling 14-3-3 to bind and resulting in reconstitution of the TEV protease activity. Const-Akt, constitutively active form of murine Akt-1. (d) Luciferase reporter assay quantifying the regulated interaction of murine Bad and 14-3-3 isoforms in NIH-3T3 cells. Cells were transfected with the indicated constructs. Mock treated cells were transfected with pcDNA3 only. Const-Akt, constitutively active form of murine Akt-1. RLUs, relative luciferase units, n = 4. (e) Renilla luciferase readings in NIH-3T3 cells show that only phosphorylated Bad influences the cellular state. Measurements were obtained from the same experiment as shown in (d). NIH-3T3 cells were transfected with the indicated constructs and the ratio of the mean value of the Renilla luciferase readings over the number of seeded cells per well (30000) were plotted.

**Figure 4:**
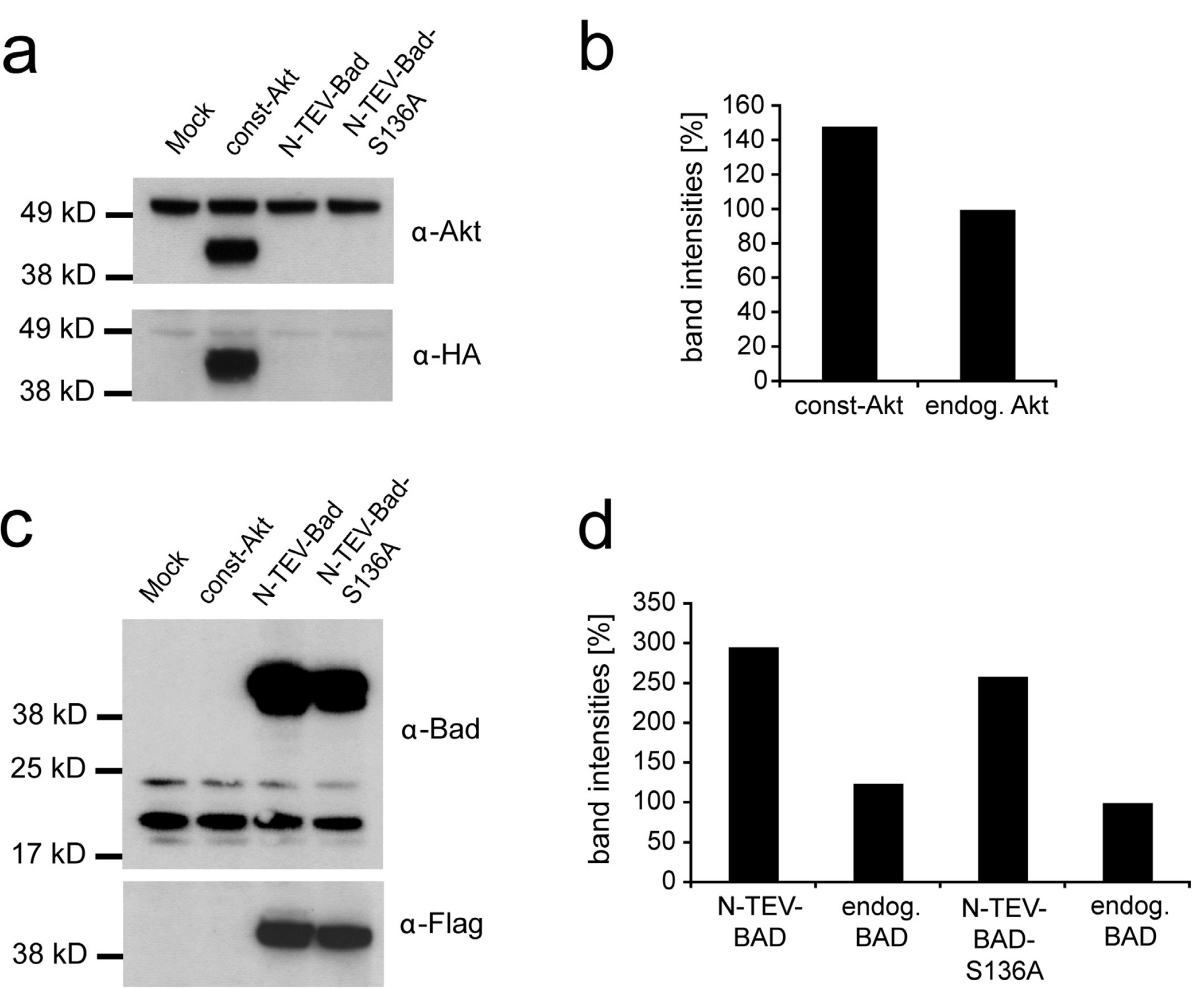
**Comparison of endogenous versus exogenous Akt and Bad protein levels**. (a, c) Western blot analyses to determine the relative amounts of endogenous protein levels of Akt (55.7 kDa) and Bad (22.1 kDa) versus the corresponding overexpressed variants. CMV-driven expression constructs coding for HA-tagged const-Akt (43.4 kDa) and the Flag-tagged N-TEV-Bad (41.0 kDa) and N-TEV-Bad-S136A (41.0 kDa) proteins were transfected in NIH-3T3 cells cultured in 6-well dishes at DNA concentrations adjusted to the amount used for the 96-well-luciferase assays. The upper panel shows that the endogenous Akt and Bad proteins as well as the exogenous fusion constructs can be simultaneously detected with α-Akt (a) and α-Bad antibodies (c), respectively. The lower panel probed with α-HA (a) and α-Flag (c) antibodies verifies the additional bands obtained with α-Akt (a) and α-Bad (c) as the exogenously introduced const-Akt, N-TEV-Bad and N-TEV-Bad-S136A proteins. (b) Relative quantification of the band intensities obtained in (a) reveals that the level of const-Akt protein is approximately 1.5 fold higher compared to endogenous Akt. (d) Relative quantification of the band intensities obtained in (c) reveals that the levels of the N-TEV-Bad and N-TEV-Bad-S136A proteins are between 3 and 2.5 fold higher compared to endogenous Bad.

Unphosphorylated Bad promotes apoptosis, however upon phosphorylation of Ser136 by Akt-1/PKB Bad binds to 14-3-3 proteins thereby inhibiting its pro-apoptotic activity [6]. To provide constant Akt-1 activity, we generated a constitutively active form of murine Akt-1 (myr-Akt-Δ4-129-HA) [24], which lacks the PH domain, but contains an N-terminal myristoylation sequence directing the kinase to the plasma membrane (Fig. 3a). Murine Bad and a mutant, which cannot be phosphorylated at Ser 136 (Bad-S136A), were fused to the N-terminal TEV fragment. Murine 14-3-3ε and 14-3-3ζ were both fused to the C-terminal TEV fragment (Fig. 3a). Western blot analysis verified the proper expression of all fusion constructs at expected molecular weights (Fig. 3b). Phosphorylation of Bad by the constitutive form of Akt-1 should facilitate the interaction of N-TEV-Bad and 14-3-3-C-TEV fusion proteins and subsequent reconstitution of the proteolytic activity of the complemented TEV protease (Fig. 3c). Upon co-transfection of constitutively active Akt-1 with N-TEV-Bad and 14-3-3ε-C-TEV or 14-3-3ζ-C-TEV signal induction ratios of 22- or 26-fold were detected using the GV-2ER reporter (Fig. 3d). When applying the cytosolic proteolysis-only reporter LucER, comparable results were obtained with slightly reduced induction levels (data not shown). Replacing Bad by the Bad-S136A mutant abolished the interaction completely, demonstrating that Ser136 is the crucial residue for the interaction (Fig. 3d) [7]. Apoptosis promoted by non-phosphorylated Bad and, conversely, survival signaling exerted by phosphorylated Bad bound to 14-3-3, may be observed [7,13]. When we normalized the readings obtained from the constitutively expressing *Renilla *luciferase constructs to the number of cells, an effect of Akt-1 and Bad on the cellular state became evident. In the presence of constitutive Akt-1 kinase activity, the *Renilla *luciferase readings were elevated by approximately 3-fold (Fig. 3e). In contrast, when Akt-1 was lacking, *Renilla *readings were lower, as evidenced in the experiment using N-TEV-Bad and 14-3-3ζ-C-TEV fusions (Fig. 3e). When the phosphorylation deficient Bad-S136A variant was used, *Renilla *firefly readings remained unaltered in the presence or absence of Akt-1. Analogous measurements were obtained using the 14-3-3ε isoform (Fig. 3e). We could also observe a several-fold increase in the relative firefly reporter activity if only both wild-type Bad and constitutively active Akt-1 were present. To determine the ratio of exogenously added Akt and Bad versus the endogenous proteins, we performed Western blots from NIH-3T3 cell lysates singly transfected with Akt or Bad. DNA amounts used for transfection were proportional to those used in the luciferase assays. Expression levels of the HA-tagged constitutively active Akt mutant over endogenous Akt was found to be approximately 1.5 fold (Fig. 4a,b). The levels of the N-TEV-Bad and N-TEV-Bad-S136A fusion proteins were approximately 3- and 2.5-fold compared to endogenous Bad (Fig. 4c, d). Transfection efficacies of NIH-3T3 cells were found to be 24 ± 2% and 21 ± 3% for EYFP-Bad and EYFP-BadS136A, respectively, based on FACS analysis (data not shown). Thus, as judged by the transfection efficiency of single plasmids, the average level of overexpression in individual cells will be increased by 5-fold. This calculation, however, can only serve as an estimation for the split-TEV assays, since up to five different plasmids are co-transfected, and most likely only a minor fraction of cells will receive a proportional amount of all plasmids. We monitored the subcellular distribution of the EYFP-Bad and EYFP-BadS136A fusion constructs by epifluorescent microscopy with and without co-transfected Akt-1 and could not detect altereded subcellular localizations (data not shown).

**Figure 5:**
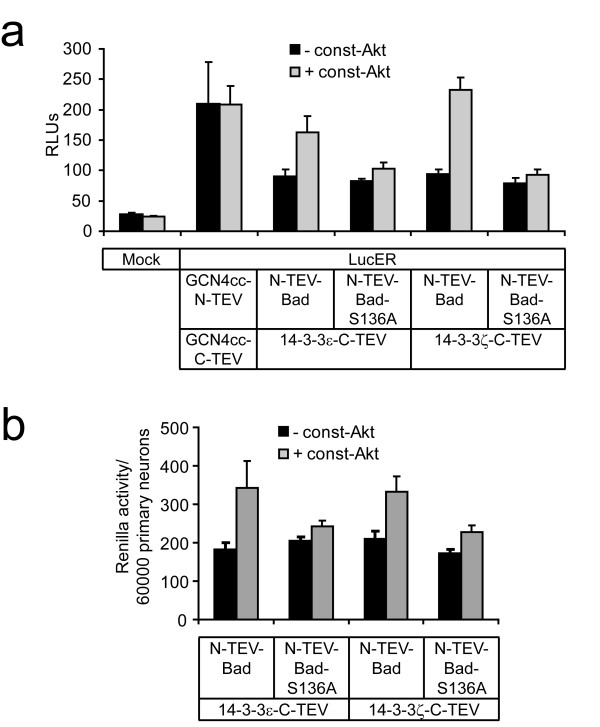
**Bad/14-3-3 split-TEV assays in primary cultured neurons**. (a) Luciferase reporter assay quantifying the regulated interaction of murine Bad and 14-3-3 isoforms in murine primary cultured cortical neurons. Cells were transfected with the indicated constructs and LucER as reporter, n = 6. (b) Renilla luciferase readings in primary cultured neurons. Measurements were obtained from the same experiment as shown in (a). The values are given as the ratio of the mean value of the Renilla luciferase readings and the number of seeded cells per well (60000).

Applying these split-TEV assays in primary cultured cortical neurons gave similar results for the Akt-1 dependent interaction of Bad and the 14-3-3ε and ζ isoforms (Fig. 5a). Our results also provide evidence for a function of these brain-enriched 14-3-3 isoforms with respect to neuronal survival (Fig. 5b).

### Monitoring Bad and 14-3-3 interactions by transiently activated endogenous Akt-1

**Figure 6:**
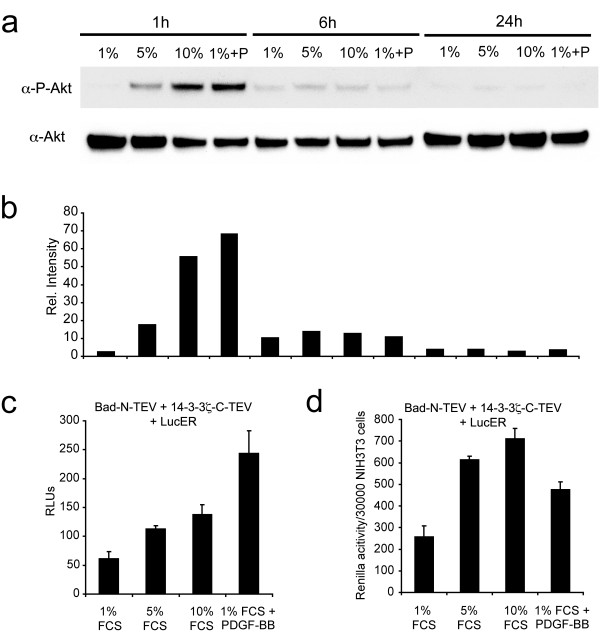
**Bad/14-3-3 split-TEV assays monitoring the effects of transiently activated endogenous Akt-1**. (a) Western blot analyzing the stimulus-dependent activated form of Akt-1. NIH-3T3 cells were treated either in 1%, 5%, 10% or 1% FCS and PDGF-BB (P). The phosphorylation of Akt-1 at residue T473 was analyzed after 1 h, 6 h and 24 h after treatment using an α-P-Akt antibody. Loading was controlled using an α-Akt antibody. (b) Relative quantification of the western blot shown in (a) showing the transient activation of endogenous Akt-1 1 h after stimulation of NIH-3T3 cells. (c) Split-TEV assay using Bad-N-TEV and 14-3-3ζ-C-TEV constructs to measure endogenous activated Akt-1. NIH-3T3 cells were treated as in (a). Analysis was performed 6 h after treatment. RLUs, relative luciferase units, n = 4. (d) Renilla luciferase readings in NIH-3T3 cells. Measurements were obtained from the same experiment as shown in (c). NIH-3T3 cells were transfected with Bad-N-TEV and 14-3-3ζ-C-TEV constructs. The mean value of the Renilla luciferase readings were given as a ratio of the values versus the number of seeded cells per well (30000).

Phosphorylation regulates Akt-1 and can be used as a surrogate marker for its activation [25]. We determined the level of endogenously phosphorylated Akt-1 in stimulated NIH-3T3 fibroblasts by Western blotting using an antibody directed against the activated, phosphorylated form of Akt-1. In parallel, we measured the interaction of Bad and 14-3-3 using split-TEV assays. Akt-1 phosphorylation was determined in extracts of either starved NIH-3T3 cells (grown in 1% serum) or upon stimulation with 5% and 10% serum or with purified PDGF-BB (Fig. 6a). As a measure of the relative level of Akt phosphorylation, we determined the pixel intensities of the resulting Western blot bands obtained for endogenous Akt and phosphorylated Akt (Fig. 4b). Peak levels of Akt phosphorylation were detected 1 h post stimulation and already substantially reduced 6 h following stimulation. Phosphorylation levels declined to baseline levels within 24 h. (Fig. 6b). In the corresponding split-TEV assay, the Bad/14-3-3 interaction was used as readout for the activated form of Akt-1. Stimulations with 5% and 10% serum and the addition of PDGF substantially increased the readout. Moreover, the increase was proportional to the relative activation of endogenous Akt-1 with PDGF-BB being the most potent stimulus (Fig. 6c). Thus, the split-TEV assay can be used to measure this phosphorylation-dependent interaction induced by the transient activity of Akt-1. Moreover, cells treated with increasing serum concentrations (5% and 10% FBS, respectively) or stimulated with PDGF-BB displayed increased *Renilla *luciferase activity (Fig. 6d). However, the increase in *Renilla *activity was markedly lower upon PDGF-BB treatment as compared to elevated serum concentrations (Fig. 6d). Therefore, the split-TEV assays are unlikely to be compromised by the stimulatory effects of the PDGF growth factor activity.

### Monitoring regulated Bad and 14-3-3 interactions with a fluorescent reporter

**Figure 7:**
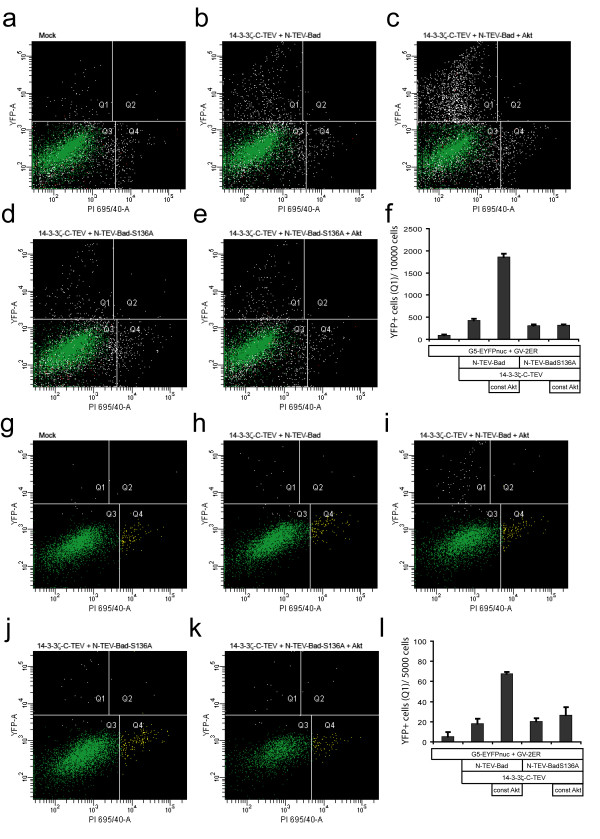
**Bad/14-3-3 split-TEV assays in HEK293 and NIH-3T3 cells using a fluorescent reporter**. (a-l) FACS analyses of HEK293 (a-f) and NIH-3T3 (g-l) cells that were co-transfected with the GV2-ER and G5-EYFPnuc reporter and the indicated expression plasmids (Mock indicates empty expression vector pcDNA3). (a-e, g-l) Representative histograms of replicate analyses with the YFP-fluorescence plotted against the PI-fluorescence intensities. Q1 gated cells display increased YFP fluorescence, Q2 gated cells are YFP/PI double-positive cells, Q3 are YFP/PI negative cell populations and Q4 represent PI positive cells. (f, l) Quantitative analysis of Q1 gated events from all replicates (n = 3) given as number of YFP-positive per 10000 (f, HEK293) or 5000 (l, NIH-3T3) cells, respectively.

Fluorescent activated cell sorting (FACS) was used to quantify the Akt-1-dependent interaction of N-TEV-Bad with 14-3-3ζ-C-TEV. Here, the readout was monitored using a nuclear targeted EYFP reporter in HEK293 (Fig. 7a–f) and NIH-3T3 cells (Fig. 7g–l). In both cell types a 3- to 4-fold increase in the number of YFP positive cells was observed, which was dependent on the presence of wild-type Bad and constitutively active Akt-1 (Fig. 7f,l). The Akt-1-mediated effect was abrogated when the N-TEV-BadS136A mutant construct was used (Fig. 7f,l). In parallel, we stained dying cells with the cell-impermeable red fluorescent nuclear dye propidium iodide (PI). Under all conditions, the number of PI-positive or PI-YFP double-positive cells were not substantially altered (Fig. 7a–e and 7g–k, and data not shown) proving that the Akt-1-mediated effects on the cellular state did not bias the split-TEV assays using the EYFP reporter.

### Monitoring adapter interactions with Nrg1-activated ErbB4 receptors

**Figure 8:**
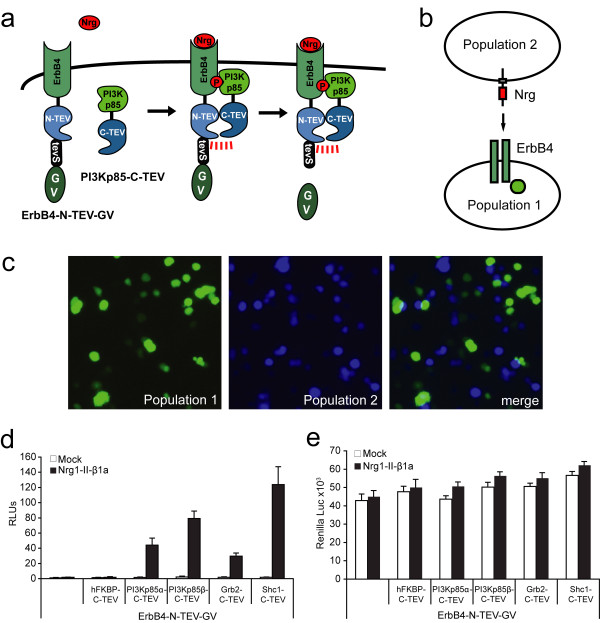
**Nrg1 induced interactions of ErbB4 and adapter proteins**. (a) Schematic drawing showing the principle of the assay. Upon Nrg stimulation, ErbB4 receptors are activated and Tyr-phosphorylated resulting in binding domains for several adapter proteins such as the PI3Kp85 subunits. In the split-TEV assays, ErbB4 is fused to N-TEV, a TEV cleavage site and the transcription factor GV (ErbB4-N-TEV-GV). The adapter proteins are fused to C-TEV (such as PI3Kp85-C-TEV). PI3Kp85 binding to ErbB4 results in the formation of a functional TEV protease made up of the N-TEV and C-TEV fragments. Proteolytic activity releases GV, which can translocate to the nucleus to induce transcription of a luciferase reporter gene. (b) Schematic drawing of the experimental setup for the 2-cell-population assay to allow ErbB4 activation exclusively at the cell membrane. The ErbB4 receptor (dark green rectangle) and the adapters (bright green circle) were transfected into population 1. The ligand neuregulin (Nrg) was transfected into population 2. This verifies that the Nrg induced ErbB4 activation and the subsequent phosphorylation-dependent interaction with the adapter protein are initiated via intercellular signaling at the cell membrane. (c) Visualisation of the 2-population assay. EYFP was co-transfected into population 1, ECFP into population 2. EYFP and ECFP were allowed to express for 20 h separately. Subsequently, both populations were mixed, and the assay was continued for additional 24 h. The merged image shows that YFP and CFP signals are not overlapping and that yellow and cyan signals are frequently in close proximity. Images were taken immediately before the luciferase assay was performed. (d) 2-population luciferase assay depicting the activation of the neuregulin-1 (Nrg1-II-b1a variant) induced phosphorylation-dependent interaction between the ErbB4 receptor and the adapter proteins PI3Kp85α, PI3Kp85β, Grb2 and Shc1. Combination of constructs were transfected into PC12 cells as indicated. RLUs, relative luciferase units; n = 6. (e) Renilla luciferase readings taken from the same assay as depicted in (d). Stimulation with Nrg1-II-b1a does not lead to substantial differences in absolute Renilla luciferase readings.

Finally, we used the well known phosphorylation-dependent interactions of cytosolic adapter proteins to Nrg1-activated ErbB4 [21] to show the applicability of split-TEV assays to monitor regulated interactions at the membrane of living cells. Therefore, ErbB4 was fused to N-TEV and the artificial transactivator Gal4-VP16 (GV) separated by a TEV protease cleavage site (tevS) (ErbB4-N-TEV-tevS-GV) (Fig. 8a). Adapter proteins (PI3K subunits α and β, Grb2 and Shc1) were fused to C-TEV fragments.

For the split-TEV assays, we expressed a Nrg-1 isoform (Nrg1-typeII-β1a) as a full-length protein [26]. To ensure that ErbB4 is specifically activated at the cell membrane, Nrg1-typeII-β1a was separately transfected into PC12 cells (population 1), whereas ErbB4, the adapters and the GV-dependent reporter were transfected into a second batch of PC12 cells (population 2) (Fig. 8b). 20 h post transfection cell populations 1 and 2 were mixed, and 24 h later the adapter/ErbB4 receptor interactions were monitored. The experimental setup of this 2-population assay was confirmed by co-expressing EYFP in population 1 and ECFP in population 2. Fluorescence microscopy of YFP and CFP revealed the existence of two separate cellular populations with non-overlapping yellow and cyan positive cells being in close contact (Fig. 8c).

Split-TEV assays show that ErbB4-N-TEV-tevS-GV interacts with the adapters PI3Kp85α-C-TEV, PI3Kp85β-C-TEV, Grb2-C-TEV and Shc1-C-TEV only if Nrg1-typeII-β1a was expressed in neighboring cells (Fig. 8d). The cytosolic protein FK506-binding protein-C-TEV fusion protein (FKBP-C-TEV) served as a negative control showing no activation in the presence or absence of Nrg1-typeII-β1a (Fig. 8d). The corresponding *Renilla *luciferase readings are highly similar between all assays showing that transfection efficiencies were similar, and that secondary stimulatory effects may have induced the assays (Fig. 8e). Thus, inter- and intracellular signaling events can be monitored with appropriately designed split-TEV assays in living cells.

## Discussion

Protein modification-dependent PPIs serve complex regulatory functions in cellular signaling cascades [27]. However, the quantitative analysis of these key cellular events still remains a challenging task. Here, we apply split-TEV assays to monitor constitutive and transient phosphorylation-dependent PPIs that occur in the cytosol and at the membrane of living cells. Thus, split-TEV assays represent an alternative option towards the goal of analysing regulated PPIs of full-length proteins within signaling cascades that occur both at the membrane and in the cytosol of living cells (this study and [5]).

Using split-TEV, we analyzed the regulated interaction of Bad with 14-3-3 isoforms in primary cultured neurons. Cellular assays in most primary cultured cells, including neurons, still represent a major challenge due to lower transfection efficiencies and limited availability. With our transfection protocol, we routinely obtained transfection efficiencies of primary cultured neurons that did not exceed 10%. Nonetheless, we were able to robustly measure the phosphorylation-dependent Bad/14-3-3 interaction in our standard 96-well format. Since observations in primary cultures may be more relevant to understand cell-type specific cellular signaling events, split-TEV assays represent a particularly valuable tool to determine modification-regulated interaction profiles in these cells. Although we mainly focused on luciferase-based reporters in this study, the fluorescent split-TEV reporter RedERnuc displayed a high signal-to-noise ratio and may represent an additional readout option when cell numbers are limiting and/or transfection efficiencies are low.

In this report, the association of the pro-apoptotic Bad and the adapter protein 14-3-3, which only binds to phosphorylated Bad, was quantified in heterologous cells and primary cultured neurons along with the effects on the cellular state evoked by the interaction itself. Although the results obtained in NIH-3T3 cells and primary cultured neurons showed the same tendency, the effect was less pronounced in primary neurons. This may be caused by the lower transfection efficiencies and/or expression levels obtained with primary neurons. In our assays, the transfection efficiencies of NIH-3T3 cells vary between 20–30%, whereas the relative number of neurons transfected is generally lower than 10%. The cell type depending differences in transfection efficiencies may also explain the results that we obtained by FACS analyses with HEK293 and NIH-3T3 cells using the fluorescent EYFPnuc reporter. Although the results between the cell types and the different reporter systems (luciferase versus fluorescence) were highly similar, the relative robustness of the readout varied. In HEK293, the Akt-1 dependent interaction of 14-3-3ζ-C-TEV with N-TEV-Bad led to an activation of the reporter in almost 20% of all cells, whereas the maximum level of activation in NIH-3T3 was below 2%. However, the relative induction ratios over controls (interaction of 14-3-3ζ-C-TEV with N-TEV-Bad without Akt-1 or with the phosphorylation mutant BadS136A variants) were comparable between cell lines. This difference could be due to variable numbers of plasmids taken up by individual cells, as up to five plasmids are required for the split-TEV assays used. In NIH-3T3 cells, split-TEV was also successfully applied to measure endogenous phosphorylation levels of Akt-1, proving the high sensitivity of split-TEV assays.

Also, we monitored phosphorylation-dependent interactions between activated ErbB4 receptor tyrosine kinases and several cytosolic adapter proteins in PC12 cells. In the presented 2-cell batch assays, ErbB4 was stimulated by a full-length Nrg1 variant expressed exclusively in neighbouring cells. The chosen isoform of Nrg-1 (Nrg1-typeII-β1a) requires proteolytic cleavage by extracellulary proteases to yield a soluble, active form [26]. We conclude that the split-TEV technique may be particularly suited to study weak and eventually transient protein interactions with a simple readout format even if complex membrane signaling is under investigation. Given the enormous complexity of mutually dependent inter- and intracellular signaling processes, the need of scalable assay systems, such as the split-TEV, is apparent. For example, the *NRG1 *gene generates at least 31 isoforms, which may have different signaling capabilities and/or different receptor affinities [19]. Nrg1 isoforms can bind to ErbB3 and ErbB4 receptors with ErbB4 having four splice variants itself. ErbB receptors can form homo- and heterodimeric complexes exerting different signaling properties, making a differential receptor/adapter association analysis in living cells a valuable and highly challenging task. We have shown that split-TEV assays can be designed to study pivotal inter- and intracellular signaling events in living cells with high sensitivity. Given its flexibility and scalability, the split-TEV technique may help to unravel the complexity of cellular signaling in the future.

## Conclusion

Split-TEV assays may complement existing techniques to study phosphorylation-dependent and transient PPIs that were induced by intrinsic kinase activities in living cells. The interactions can be monitored with full-length cytosolic proteins in heterologous cell lines as well as in primary cultured neurons. All assays were performed in multi-plate formats and could therefore be adapted to higher throughput to screen for pharmacological substances interfering with pivotal PPIs within cellular signaling cascades.

## Methods

### Construction of Expression Plasmids

Expression plasmids were constructed with PCR techniques using proofreading polymerases (Pfu and EasyA, Stratagene). PCR products were either TA-overhang sub-cloned in pGEM-T (Promega) using standard molecular cloning procedures or were modified with attB1/2 recombination sites for the 'Gateway' recombination cloning (Invitrogen). All protocols were performed according to the manufacturers instruction (Invitrogen). Reporter plasmids used have been described recently [5].

### Western Blotting of Expression Constructs

COS1 cells were transfected with 5 μg of plasmid DNA per sample, cultured for 40 h and lysed in RIPA buffer (50 mM Tris-HCl pH 7.4, 150 mM NaCl, 1 mM EDTA, 1% Triton X-100, 1% sodium deoxycholate, 0.1% SDS), including protease inhibitors (Complete kit, Roche). The lysates were sonicated and the cell debris was removed by centrifugation. Equal amounts of lysate containing LDS sample buffer were loaded on precasted gels (Invitrogen) and blotted on PVDF membranes (Amersham Biosciences). The membranes were blocked in blocking buffer (5% milkpowder in TBS-T) and probed with primary (αFlagM2 1:5000 in blocking buffer, Sigma; αHA 1:1000, Roche) and secondary antibodies (HRP goat-α-mouse 1:5000, HRP goat-α-rabbit 1:5000, HRP goat-α-rat 1:1000, Jackson Immuno Research Labs). Anti-phospho-Akt (Ser473) (Cell Signaling Technology, #9272, 1:1000), anti-Akt-1 (Cell Signaling, #9271, 1:1000) and anti-Bad antibodies (Cell Signaling, #9292, 1:1000) were used to determine exogenous, activated and total amounts of Akt-1 and Bad in transfected and stimulated NIH-3T3 cells. For luminescence detection, the Western Lightning kit (Perkin Elmer Life Science) was used and ECL-films (Amersham) were developed in a Kodak X-OMAT 1000. Band intensities of endogenous and transfected proteins were determined using ImageJ 1.37. Scanned images were computationally inverted, the bands in question were equally boxed, and the intensities were measured and converted to mean values. For comparison, the band intensities representing the endogenous protein were set to 100% and plotted as histogram.

### Cultivation and Transfection of Cell Lines

NIH-3T3, COS1 and PC12 cells were cultured in DMEM with 1 g/l glucose (BioWhittaker) and supplemented with 5% fetal calf serum (FCS, Invitrogen) (NIH-3T3 and COS1 cells) or 10% FCS and 10% horse serum (HS, Invitrogen) (PC12 cells) and 200 mM Glutamax (Invitrogen). For luciferase experiments, NIH-3T3 cells were cultured in 1% FCS and analysed 24 h (using proteolysis-only reporters) or 24 to 48 h (using 'transcription-coupled' reporters) post transfection. In 2-population cell assays, PC12 cells were cultured with 5% FCS and 5% HS. Therefore, PC12 cells were separated in two populations and transfected with plasmids encoding for ligands only (population 1) or membrane and cytosolic proteins along with reporters (population 2). Populations 1 and 2 were allowed to individually express the proteins for 20 h. Subsequently, the cells were washed, the populations were mixed and the final assay was performed 24 h later. Cells were transfected with Lipofectamine2000 (Invitrogen) according to the manufacture's instructions. For specific stimulation of the PI3K-Akt pathway, NIH-3T3 cells were treated with PDBF-BB (50 ng/ml, PeproTech).

### Preparation and Transfection of Primary Neurons

Primary neurons were prepared from the cortex and hippocampus of E17 old mouse embryos. After preparation of the hippocampus and the cortex, both tissues were incubated in HBSS (supplemented with 10 mM Hepes, NaHCO_3 _and 1× penicillin/streptomycin) containing 0.5× trypsin-EDTA and 0.1 mg/ml DNAseI for 10 min at 37°C. The cells were further dissociated by gentle trituration until a homogenous dispersion was achieved. The cells were pelleted (800 rpm for 10 min) and resuspended in Neurobasal medium supplemented with B27-supplement (1:50, Gibco), L-glutamine (1:100, 2 mM), penicillin (100 U/ml), streptomycin (100 μg/ml), MK-801 (10 μM, Tocris), NGF (0.1 ng/μl, Promega) and β-FGF (0.1 ng/μl, PeproTech). Finally, cells were counted and plated for the luciferase assays (96-well plate: 6 × 10^4^/well). For transient transfections, the primary neurons were cultured for 3 to 4 days, followed by the removal of 60% of the medium, and the addition of the Lipofectamine2000/plasmid mix. Six hours later, 75% of the medium was removed, and replaced by fresh medium and analysed 24 h post transfection.

### Luciferase Measurements

Luciferase assays were performed in 96-well plates using the Dual-Luciferase kit (Promega). For standardization of transfection variability and increased readout stability, a mix of plasmids coding for renilla-luciferase (RL) was used (CMV-RL, TK-RL and SV40-RL; 1:2:10 molar proportions, respectively). DNA amounts were adjusted to equal levels with pcDNA3 or pCMV-EYFP. Cells were treated with lysis reagent (Promega) according to the manufacturers instructions. Coupled firefly and Renilla-luciferase assays were performed in a Lumat LB96V reader (Berthold Technologies). Results were given as Renilla-normalized relative luciferase units (RLUs) ± standard deviation (SD). All experiments were repeated at least three times with a minimum of three replicates for each data point.

### FACS Analysis

For FACS analysis, HEK293 and NIH-3T3 cells were seeded on day 1 in 24 well plates with 3 × 10^5 ^and 10^5 ^cells per well, respectively. On day 2, cells were transfected with expression constructs along with the GV2ER plasmid and a nuclear targeted Gal4-dependent EYFP reporter (G5-EYFPnuc) using Lipofectamin 2000 (Invitrogen). 30 h later, cells were stained for 30 min with the nuclear dyes Hoechst 33342 (Hoe, cell permeable, labels all cells) and Propidium Iodide (PI, cell impermeable, labels dying cells) (Invitrogen) according to manufacturer specifications. Single cell suspensions were prepared by trypsin treatment and thorough pipetting in PBS containing 5 mM EDTA. Before FACS analysis, cells were filtered through 50 μm nylon meshes and kept on ice. Hoe, YFP and PI analyses were performed with standard filter settings, gates were adjusted with negative and positive control samples. All experiments were performed with three independent replicates for each data point. Results were given as numbers of YFP-positive cells per 10000 (HEK293) or 5000 (NIH-3T3) Hoe-positive stained cells. FACS measurements were performed with a FACS-Aria (Becton-Dickinson). Errors are given as SEM.

## Authors' contributions

MCW and MJR designed the study and wrote the manuscript. MCW and LR performed the experiments. AB was involved in the cloning of adapter constructs. All authors read and approved the final manuscript.
